# P33 Supporting safe and confident use of the updated intra-abdominal antimicrobial guideline at Leeds Teaching Hospitals NHS Trust

**DOI:** 10.1093/jacamr/dlag102.039

**Published:** 2026-06-26

**Authors:** Samuel Foley, Sarah Chadwick

**Affiliations:** Leeds Teaching Hospitals NHS Trust, Leeds, UK; NHS West Yorkshire ICB, Leeds, UK

## Abstract

**Background:**

Intra-abdominal sepsis (IAS) guidelines at LTHT were updated in December 2025, secondary to rising antimicrobial resistance seen in intra-abdominal *E.coli* samples within West Yorkshire. This move also aligned with the UK’s National Action Plan for antimicrobial resistance, to increase proportion of antibiotic use from the ‘Access’ category. This guideline change saw the removal of ‘Watch’ antibiotics, cefuroxime, piperacillin/tazobactam and co-amoxiclav, and subsequent addition of empirical ‘Access’ antibiotics, gentamicin and co-trimoxazole. Multidisciplinary team feedback, incident reports and anecdotal evidence within our abdominal medicine and surgery (AMS) clinical service unit (CSU) highlighted gaps in knowledge and low confidence surrounding the prescribing and monitoring of these complex antimicrobials. Gentamicin and co-trimoxazole are recommended as first line empirical agents for severe and non-severe sepsis at LTHT as of December 2025, with both antimicrobials providing challenges for prescribers working within this CSU.

**Objectives:**

To identify barriers limiting the correct and confident application of the updated IAS guideline at LTHT.

**Methods:**

A qualitative survey was developed using the COM-B model and disseminated to prescribers working within the AMS CSU in person and via email. The online survey was developed using Microsoft Forms and remained open for a period of 2 weeks. A 5-point Likert scale was used throughout, where responses to questions ranged from ‘strongly disagree’ to ‘strongly agree’. An opportunity for free-text comments was provided the end of the survey. Responses were mapped to the Theoretical Domains Framework (TDF) and COM-B.

**Results:**

A total of 33 responses were collected with respondents ranging from consultants, resident doctors, non-medical prescribers (NMPs) and prescribing pharmacists. Physical opportunity played a key role within application of the guideline, with many respondents stating that time pressures did not allow them to apply the guidelines as intended. Similarly, many stated that the electronic prescribing system at LTHT did not support safe use of the recommended agents. Free-text responses compounded these findings, with respondents highlighting that trust specific resources are not easily accessible and lack clarity. Many prescribers did not feel comfortable applying the guideline in complex patients and were not reassured by the safety of gentamicin, highlighting reflective processes as another barrier. Resident doctors, NMPs and prescribing pharmacists scored similarly across domains, with consultants scoring positively across all domains, albeit with the smallest sample size. Prescribers felt confident in applying IV to oral switches when appropriate, knowing what treatment to use in clinically improving patients and when to seek specialist advice or senior support.

**Conclusions:**

Implementation of improvements to current guidelines and prescribing protocols at LTHT are high priorities following thematic analysis of the COM-B survey. Further education is required for prescribers regarding application of the IAS guidelines, and the sharing of positive antimicrobial success stories is necessary to reassure and change prescribing culture associated with gentamicin.

